# SIMULATION IN AGE SUIT: AN INNOVATIVE NURSING EDUCATION INTERVENTION TO INCREASE THE UNDERSTANDING OF OLDER ADULTS

**DOI:** 10.1093/geroni/igad104.2121

**Published:** 2023-12-21

**Authors:** Catharina Gillsjö (Gillsjo), Björn Bouwmeester Stjernetun, Jenny Hallgren

**Affiliations:** University of Skövde, Skövde, Vastra Gotaland, Sweden; University of Skövde, Skövde, Vastra Gotaland, Sweden; University of Skövde, Skövde, Vastra Gotaland, Sweden

## Abstract

**Background:**

Negative attitudes towards older adults are often recognized in society, especially among health care providers. Lack of education in gerontology and geriatrics and limited practical training in nursing education have been identified as influencing aspects. Simulation in education through age suit has been recognized to increase empathy and reduce negative attitudes towards older adults. An innovative education intervention with simulation in age suit with a follow up reflection seminar was developed to counteract ageism and increase the understanding of aging, age related health problems, older adults and to influence the willingness to provide care to older adults.

**Methods:**

The intervention with simulation in age suit, grounded in the pedagogy experiential learning, was implemented in the fourth semester in the three-year nursing education program. The simulation was conducted in a highly accessible home environment equipped with welfare technology and other technical aids. The nursing students were assigned a persona, a person with different health problems, and performed scenarios consisting of daily chores. Research on nurses’ experiences of the intervention and its sustainability through the education program and on perceptions of providing care for older adults, is conducted qualitatively and quantitatively in a doctoral project.

**Conclusion:**

The learning acquainted through simulation in age suit became embodied both physically and psychologically. The simulation in age suit conveyed new insights as need of time and patience. The technology in the environment both hindered and facilitated activities in daily life. Implications suggested the need for all health and social care providers to carry out this simulation.

